# Development and Implementation of a Mobile Phone–Based Prevention of Mother-To-Child Transmission of HIV Cascade Analysis Tool: Usability and Feasibility Testing in Kenya and Mozambique

**DOI:** 10.2196/13963

**Published:** 2019-05-13

**Authors:** Nami Kawakyu, Ruth Nduati, Khátia Munguambe, Joana Coutinho, Nancy Mburu, Georgina DeCastro, Celso Inguane, Andrew Zunt, Neil Abburi, Kenneth Sherr, Sarah Gimbel

**Affiliations:** 1 Center for Global Health Nursing University of Washington Seattle, WA United States; 2 Department of Global Health University of Washington Seattle, WA United States; 3 Network of AIDS Researchers in East and Southern Africa Nairobi Kenya; 4 Department of Paediatrics and Child Health University of Nairobi Nairobi Kenya; 5 University of Eduardo Mondlane Maputo Mozambique; 6 Manhiça Health Research Centre Manhiça Mozambique; 7 Health Alliance International Beira / Chimoio Mozambique; 8 Paul G Allen School of Computer Science and Engineering University of Washington Seattle, WA United States; 9 Health Alliance International Seattle, WA United States; 10 Department of Family and Child Nursing University of Washington Seattle, WA United States

**Keywords:** mHealth, quality improvement, engineering, HIV, mother to child transmission, implementation science

## Abstract

**Background:**

Prevention of mother-to-child HIV transmission (PMTCT) care cascade failures drive pediatric HIV infections in sub-Saharan Africa. As nurses’ clinical and management role in PMTCT expand, decision-support tools for nurses are needed to facilitate identification of cascade inefficiencies and solutions. The mobile phone–based PMTCT cascade analysis tool (mPCAT) provides health facility staff a quick summary of the number of patients and percentage drop-off at each step of the PMCTC care cascade, as well as how many women-infant pairs would be retained if a step was optimized.

**Objective:**

The objective of this study was to understand and improve the mPCAT’s core usability factors and assess the health workers’ experience with using the mPCAT.

**Methods:**

Overall, 2 rounds of usability testing were conducted with health workers from 4 clinics and leading experts in maternal and child health in Kenya and Mozambique using videotaped think aloud assessment techniques. Semistructured group interviews gauged the understanding of mPCAT’s core usability factors, based on the Nielsen Usability Framework, followed by development of cognitive demand tables describing the needed mPCAT updates. Post adaptation, feasibility was assessed in 3 high volume clinics over 12 weeks. Participants completed a 5-point Likert questionnaire designed to measure ease of use, convenience of integration into work, and future intention to use the mPCAT. Focus group discussions with nurse participants at each facility and in-depth interviews with nurse managers were also conducted to assess the acceptability, use, and recommendations for adaptations of the mPCAT.

**Results:**

Usability testing with software engineers enabled real-time feedback to build a tool following empathic design principles. The revised mPCAT had improved navigation and simplified data entry interface, with only 1 data entry field per page. Improvements to the results page included a data visualization feature and the ability to share results through WhatsApp. Coding was simplified to enable future revisions by nontechnical staff—critical for context-specific adaptations for scale-up. Health care workers and facility managers found the tool *easy to use* (mean=4.3), used the tool *very often* (mean=4.1), and *definitely intended to continue to use* the tool (mean=4.8). Ease of use was the most common theme identified, with emphasis on how the tool readily informed system improvement decision making.

**Conclusions:**

The mPCAT was well accepted by frontline health workers and facility managers. The collaborative process between software developer and user led to the development of a more user-friendly, context-specific tool that could be easily integrated into routine clinical practice and workflow. The mPCAT gave frontline health workers and facility managers an immediate, direct, and tangible way to use their clinical documentation and routinely reported data for decision making for their own clinical practice and facility-level improvements.

## Introduction

### Background

Gaps in the implementation of prevention of mother-to-child HIV transmission (PMTCT) services drive pediatric HIV infection in sub-Saharan Africa (SSA) [1-3]. Although PMTCT studies in SSA have been shown to reduce HIV transmission rates among breastfed infants to as low as 1% to 3% [4,5], the rates are several times higher in practice because of the high drop-out rates of HIV-positive women along the PMTCT care cascade [1-3,6-9]. Barriers to PMTCT service uptake and retention include lack of coordinated and continuous care for HIV-positive mother-baby pairs [10-12]; only 50% of infants born to HIV-positive women in Kenya and Mozambique are tested for HIV within the first 2 months of life [13,14].

To simplify the cascade and improve PMTCT services, the World Health Organization introduced Option B+ in 2013 by which HIV-positive women initiate lifelong antiretroviral therapy (ART) during pregnancy regardless of CD4 count [15]. Since then, studies have found Option B+ to be cost-effective [16-19]. A study in Malawi found that Option B+ not only prevented infant infections, but also increased the mother’s 10-year survival rate by more than 4-fold compared with the standard of care [19]. There are also multiple health system challenges to implementing Option B+. The most common among them are health facility resource limitations such as shortage of staff and drugs [10,20,21]. Nurse staffing and workloads are strongly correlated with pregnant women completing steps along the complex cascade of Option B+ services [22]. Consequently, understaffing and undertraining of nurses lead to long wait times, inefficient patient flow, gaps in patient tracking [20,21], and thus, suboptimal adherence and retention of HIV-positive women [23-25]. Given that the quality of Option B+ services is dependent on specific health facility characteristics [22], interventions to improve services should be adaptable to the specific health facility’s context.

The systems analysis and improvement approach (SAIA) is a 5-step process using systems engineering theory to guide facility-level staff and managers to maximize effectiveness of PMTCT services [26]. The first step is to improve the understanding of the inefficiencies by conducting a cascade analysis using the PMTCT cascade analysis tool (PCAT), which provides a systems-level view to track patient flow through the PMTCT cascade. This is followed by value stream mapping of the cascade to guide identification and prioritization of modifications addressing workflow inefficiencies. Steps 3 to 5 use continuous quality improvement (CQI) to iteratively test and modify facility-level improvement strategies. The SAIA intervention increased antiretroviral coverage by 3-fold and screening of HIV-exposed infants (HEI) by 17-fold in Kenya and Mozambique [27]. However, the PCAT’s usability was inhibited by low computer availability and literacy at the health facilities [28]. As such, use of the PCAT was led by study nurses rather than facility personnel. Subsequently, the SAIA team developed and beta tested a mobile phone–based app of the PCAT (mPCAT) to facilitate its independent use by frontline facility staff.

Mobile phones have become widely available in SSA, with 73% of the population estimated to have a mobile cellular subscription [29]. Mobile phone systems for surveillance and data collection have been found to improve data quality [30-32] and be more time- and cost-efficient [30,32-35] than the traditional pen-and-paper method. The accessibility and literacy of mobile phones in SSA makes it an advantageous platform for scale-up and provides an opportunity for facility-level health care staff to use it for data-driven decision making.

### Objectives

The objective of this paper was to report the process of adapting the beta-tested version of the mPCAT app through usability testing in Kenya and Mozambique and share findings from feasibility testing conducted to examine the acceptability and fit of the mPCAT as part of the broader SAIA intervention.

## Methods

The usability and feasibility testing were conducted with nurses, health facility support staff, facility managers, and PMTCT experts between March and December 2017 in Kenya and Mozambique to understand and improve the mPCAT’s core usability factors and assess health workers’ experience with using the mPCAT.

### Setting

Sites for usability and feasibility testing were high-volume public facilities that provided a full range of PMTCT services and selected for inclusion based on experience with Option B+ service delivery and experience with SAIA, including the Microsoft Excel-based PCAT. A total of 2 sites in Kenya, one in the capital city of Nairobi and the other in the coastal city of Mombasa, participated in usability testing. In Mozambique, 2 sites, one in the capital city of Maputo and the other in Chimoio, the capital of Maniça Province in Central Mozambique, participated in usability testing. For feasibility testing, 2 sites in Nairobi and 1 site in Maputo were selected. The 2 sites in Kenya were selected as there was particularly high enthusiasm in Kenya in using the mPCAT tool, especially among health managers. Human resources for health are much more constrained in central Mozambique; thus, feasibility testing in more than 1 facility was not possible.

#### Kenya

Kenya adopted Option B+ in 2014. An estimated 76% of HIV-positive pregnant women in Kenya received ARTs for PMTCT in 2017 [14]. Transmission rates are estimated to be 5% at 6 weeks [36] and between 8% to 15% when including the breastfeeding period [9,36]. Only 51% of HEIs receive virological testing within the first 2 months of life [13].

#### Mozambique

Although Mozambique has made impressive achievements in increasing PMTCT coverage to 95%, it is challenged by high drop-out rates ranging from 32% loss to follow-up in Northern Mozambique [23] to only 5% to 30% of women returning for pharmacy refills at 90 days in Central Mozambique [20]. Owing to this high drop-out, mother-to-child HIV transmission rates double from 3% at 6 weeks to 6% at the end of the breastfeeding period [36]. Only half of HEIs receive virological testing within the first 2 months of life [13].

### Intervention

The objective of the mPCAT is to facilitate facility-level staff and manager’s rapid, independent, and quantitative tracking of patient flows through the PMTCT cascade. Staff enter routinely collected facility data and the tool calculates the number and proportion of women and children flowing through each step of the PMTCT cascade, broken down into the flow from antenatal care (ANC) through birth and the subsequent postpartum period. The tool calculates the number lost at each step and estimates the additional number of women and HEIs who would complete all steps of the PMTCT cascade, if each step was optimized. The results are reviewed by the health facility staff to prioritize improvements by cascade steps to optimize overall PMTCT services using CQI tools as part of the SAIA.

### Usability Testing

Usability testing was conducted between March and June 2017 to examine how well the mPCAT functions and assess user experiences of the app’s learnability, efficiency, memorability, error recovery, and satisfaction [37]. Usability testing is an important part of designing effective mobile health (mHealth) tools that are easy to use [37], increasing the likelihood for adoption of mHealth projects [32,33,38].

#### Participants

A total of 12 nurses were purposively selected from study sites to ensure representation across the PMTCT care cascade (ANC, maternity, and children at-risk care). Furthermore, 11 leading experts in maternal and child health in Kenya and Mozambique, including those from the Ministry of Health, University of Eduardo Mondlane, and University of Nairobi, were also purposively selected for usability testing. The sample size was based on previously published development and feasibility studies testing mobile phone technology to promote behavior change [39,40].

#### Data Collection

At the start of usability testing, participants were reoriented to the SAIA and introduced to the mPCAT by 2 study facilitators. Software developers were also present during usability testing to observe the participants’ use of the mPCAT app. Participants were then given instructions to perform a series of sample tasks during videotaped think aloud exercises [37], where participants were asked to give their impression of the task, including what was difficult, what questions users had, and any feedback on improvements. Tasks included downloading the app, navigating through the app, and entering and interpreting data. At the end of each meeting, in-depth semistructured group interviews were conducted by 2 data collectors to explore what users found difficult with navigation, in entering data, interpreting data, and their preferences for the appearance of the app, such as its color scheme, graphics, and labels. Nielsen Usability Framework of learnability efficiency, memorability, error recovery, and satisfaction [37] guided the development of the think aloud exercises, as well as the interview guide.

#### Data Analysis

A codebook was developed through iterative coding of the videotaped think aloud exercises in Microsoft Excel guided by Nielsen Usability Framework. This codebook was then applied and revised through coding of in-depth group interview transcripts. Coding was completed independently and concurrently by 2 staff members in Kenya and 2 staff members in Mozambique. Coding was discussed with the principal investigator, who acted as a facilitator and tiebreaker. Codes were organized into usability themes and then incorporated into a cognitive demands table, a method to synthesize data such that the findings can be directly applied to inform product improvements [41]. The cognitive demands table headings used in the analysis were mPCAT task, description of difficult cognitive element, reason for difficulty, missing knowledge or skills affecting task, and potential cues or strategies that can be used to ease the task. The findings from the usability test informed updates to the mPCAT app, which was used for feasibility testing.

### Feasibility Testing

Feasibility testing was conducted from September to December 2017 to assess whether the adapted mPCAT could be successfully used by health care workers engaged in Option B+ service delivery as part of their system analysis and improvement efforts. The feasibility domains of interest included domains defined by Bowen et al as acceptability, implementation, and adaptation [42]. Feasibility studies are critical for the development of mHealth interventions to ensure smooth integration into health systems and successful scale-up [32,33].

#### Participants

Participants for feasibility testing were purposively selected from study sites based on prior experience working in Option B+ and representation across the PMTCT care cascade (ANC, maternity, children at-risk care). A total of 16 nurses, nurse managers, and other health facility support staff, such as counselors working in Option B+ services, participated in the questionnaires for feasibility testing. Furthermore, 10 nurses and health facility support staff participated in the focus group discussions (FGDs) and 3 nurse managers, 1 each from the study sites, were selected to participate in the in-depth interviews. The sample size was determined based on previously published feasibility studies testing mobile phone technology to promote behavior change [39,40].

#### Data Collection

Feasibility testing included a baseline visit by 2 study staff to reorient participants to the SAIA and introduce participants to the mPCAT and follow-up visits at 2, 4, 8, and 12 weeks post baseline to monitor and support implementation. The final visit included a 5-point Likert questionnaire designed to measure ease of use, convenience and integration into work, and future intention to use the mPCAT. FGDs with nurse participants at each facility and in-depth interviews with nurse managers were also conducted by 2 study staff to assess the acceptability, use, and recommendations for adaptions of the mPCAT.

#### Data Analysis

Descriptive statistics of the questionnaire data was calculated using R Studio (version 1.0.153). A codebook was developed through iterative coding of transcripts from FGDs and in-depth interviews using ATLAS.ti (version 8). Codes were guided by Bowen et al’s feasibility framework [42]. One study staff and the principal investigator concurrently and independently coded 2 transcripts and then discussed and revised the codebook. This codebook was then applied to the remaining 4 transcripts and revised and reapplied to all transcripts until no revisions to the codebook was necessary. Once coding was finalized, codes were grouped into major themes guided by the feasibility framework and exemplary quotations were identified to represent the themes.

### Ethical Approval for Usability and Feasibility Testing

The study procedures were reviewed and ethics approval obtained from the Institutional Review Board at the University of Washington (number 51026). The ethics review board of Kenyatta National Hospital and University of Nairobi in Kenya (P221/03/2016), as well as the ethics review board of the University of Eduardo Mondlane in Mozambique (CIBS FM&HCM/49/2016) approved the study. Furthermore, administrative approval was obtained from the Mozambican Ministry of Health. Written consent was obtained from all participants. No unique identifiers were collected, and the importance of maintaining confidentiality was emphasized during training of data collectors and at the start of group interviews and FGDs. Study data were stored in password-protected files that were only available to study staff for the purpose of data analysis.

## Results

### Usability Testing

#### Data Entry Interface of the Mobile Phone–Based Prevention of Mother-To-Child HIV Transmission Cascade Analysis Tool: Strengths, Challenges, and Improvements

Participants described 2 main challenges with data entry in mPCAT. First, participants had difficulty understanding what data should be entered into the mPCAT. This was partly because of the differences in terminologies used in the national data collection forms and the initial mPCAT. For example, in Kenya, *previously identified HIV+* is reported in the national forms as *known positive on entry to ANC*.

To address this, terminologies used for data entry labels were revised to match national forms. In addition, toggling functionality was introduced in the Kenya version so that users could toggle over the labels and they could be shown which form and data variable point should be used to input data. Data points are not enumerated on national forms in Mozambique; therefore, this feature was not added for the Mozambique version.

Participants also had trouble with navigating the mPCAT app. The original version of the app opened directly to the data entry page with multiple textboxes without an explanation or a home screen to orient the unfamiliar user. Participants also found it challenging to scroll up and down a page to enter multiple data entry fields on 1 page and expressed a preference for scrolling left to right. Many participants had difficulty finding the *Next* button and took many tries to advance to the next screen. The *Back* option was similarly difficult for participants to find. As a result, most users thought that there was no option beyond starting over, if they accidentally advanced to the next screen or made a data entry error in a previous screen. Another design challenge identified in the initial app was that, because there was no *Save* feature, users needed to complete data entry in 1 sitting without interruption. In usability testing, participants were also observed skipping data entry fields, which led to errors in the results.

**Figure 1:**
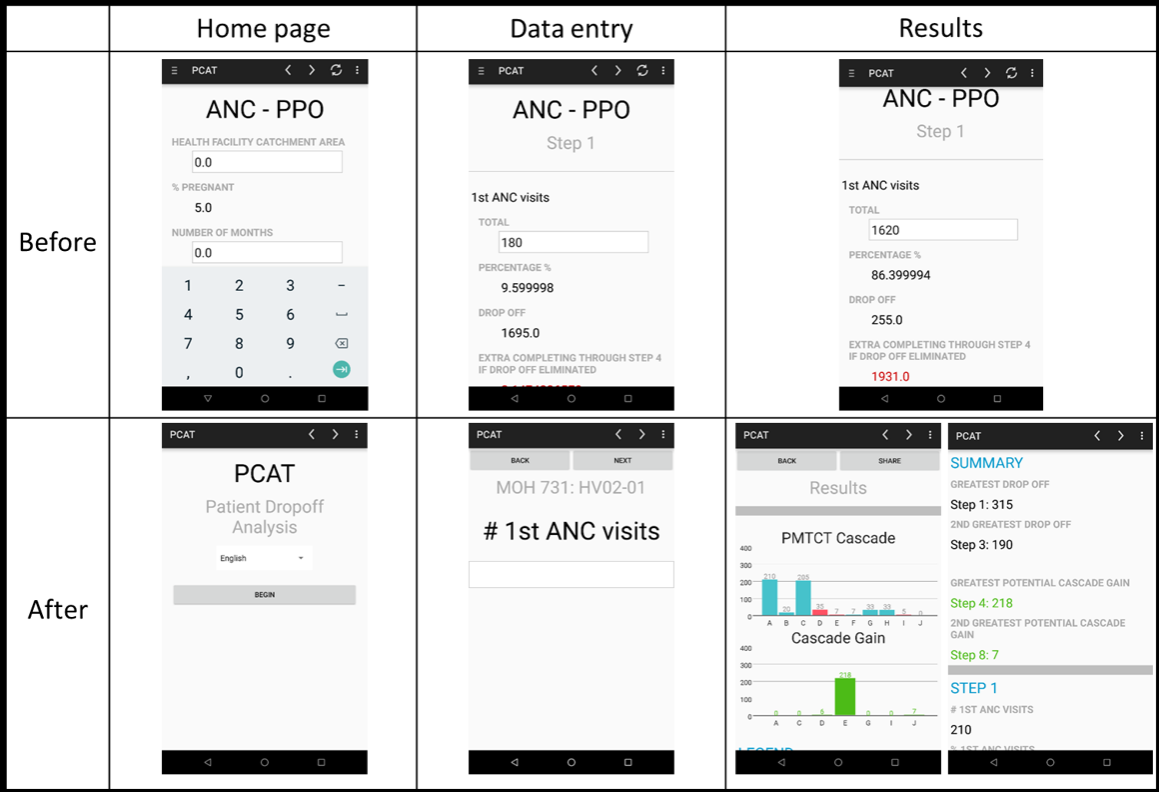
Screenshots of home page, data entry page, and results page before and after mobile phone–based prevention of mother-to-child HIV transmission cascade analysis tool usability testing. ANC: antenatal care; MOH: Ministry of Health; PCAT: prevention of mother-to-child HIV transmission cascade analysis tool; PMTCT: prevention of mother-to-child HIV transmission; PPO: prophylaxis.

To address these issues, a home page was added to orient the user to the app (Figure 1). Navigation was changed so that each page only had 1 data entry field and users had to answer the item before being able to advance to the next page (Figure 1). A clearly visible *Back* button at the top of the screen was added along with an adjacent *Next* button. The option to save and exit was also added so that users could return at a later time to enter data or retrieve reports. The large font size in the first version of the mPCAT was well received by users, many of who needed but did not wear glasses, and preserved.

#### Data Visualization and Summary Results Interface of the Mobile Phone–Based Prevention of Mother-To-Child HIV Transmission Cascade Analysis Tool: Strengths, Challenges, and Improvements

Participants had difficulty viewing and interpreting the numeric results displayed on the same page as the data entry page. On some pages with multiple data entry fields, users had to scroll down to view the results. As the results were displayed at each step of the PMTCT cascade, users would have to navigate between each page to compare the results, making it difficult to identify the greatest drop-off and optimization point. To address this, a summary results page with 3 component parts were added. One was a data visualization feature to graphically present the results and another, a summary of where the greatest drop-off in the cascade occurs and where the greatest potential is for gains. Third, the numeric results were color coded where red indicated losses and green indicated gains.

Participants also requested a feature on the app so that the results could be exported to print or shared with others. As such, a feature to export the results was added, including the ability to share the results through WhatsApp. Though the results could also be shared by email, participants expressed a preference for sharing results through WhatsApp, as it was a commonly used tool by nurses and other health care staff.

### Feasibility Study

#### Acceptability of the Mobile Phone–Based Prevention of Mother-To-Child HIV Transmission Cascade Analysis Tool Among Users

Acceptability is the extent to which those delivering and receiving the strategy find it appropriate and satisfying [42]. Overall, users found the mPCAT app easy to use (mean=4.19, SD 0.98), reported to use it regularly during the study period (mean=4.00, SD 1.16), and intended to use the app in the future (mean=4.75, SD 0.58; Table 1). On average, responses were 1.1 points higher among participants in Mozambique than in Kenya.

**Table 1:** The mean (SD) of participant acceptance of mobile phone–based prevention of mother-to-child HIV transmission cascade analysis tool (N=16).

Participant acceptance	Kenya (n=10), mean (SD)	Mozambique (n=6), mean (SD)	Total (N=16), mean (SD)
Ease of use	3.70 (0.95)	5.00 (0)	4.19 (0.98)
Regular use	3.40 (1.08)	5.00 (0)	4.00 (1.16)
Intent to use	4.60 (0.70)	5.00 (0)	4.75 (0.58)

A common theme expressed by participants during FGDs and in-depth interviews (IDIs) was how easy the mPCAT app was to use, both for entering data and in interpreting the results. With regard to ease of data entry, one participant explained:

The mPCAT is a very easy app, it is not complicated and the questions are easy to understand. It was really easy using the mPCAT.

FGD, Staff E

With regard to ease of data interpretation, one participant expressed how the new data visualization feature added to the mPCAT after usability testing improved data interpretation:

The previous mPCAT that we were using, it was not as easy as the one we are using now. Because the other one isn’t giving us a bar graph, but the one that we’re using now, it is able to give you the conclusion in the form of a graph. You are able to see easily, this is where we need to improve, this is what we are doing good.

FGD, Staff L

Participants emphasized how the mPCAT made data interpretation easy and accessible, and how this facilitated data-driven decision making:

With the mPCAT tool, you were able to see your gaps immediately. And you are able to do your interventions immediately. So, it’s a tool that I would recommend because it gives you instant, instant results.

IDI, Manager A

One example of a gap identified by users of the mPCAT was the low rate of CD4 testing among HIV-positive women. The mPCAT flagged that the facility was systematically missing CD4 testing of HIV-positive women, leading the facility to track women with missed CD4 tests and increase awareness among providers of this service delivery gap.

#### Implementation of Mobile Phone–Based Prevention of Mother-To-Child HIV Transmission Cascade Analysis Tool in Health Facilities

Implementation is the extent to which a strategy can be delivered in a defined but uncontrolled context [42]. All 3 study facilities reported using the mPCAT and there was consensus among staff that leadership, such as the facility-in-charge, was supportive of implementation. There was variability among FGD participants on the perceived effect of mPCAT on workload. One participant responsible for data analysis and reporting expressed that it decreased their workload by streamlining workflow. Participants in another facility described how using the mPCAT and making system changes increased their workload as once inefficiencies were identified, strategies needed to be adapted to rectify the challenges. Participants in a third facility found that the mPCAT neither increased nor decreased their workload, as the initial learning phase increased the workload but the improvements resulted in more efficient services, thereby reducing the workload.

Participants expressed that mPCAT, as part of the SAIA intervention, helped improve communication and team building across different services such as ANC and laboratory. As one participant explained:

There has been a great improvement between the teams. We have jointly identified the problems and interventions.

FGD, Staff I

Another participant elaborated:

It really improved our communication. Before, we didn’t speak as a team. We only discussed these issues rarely, but after [starting SAIA] we discussed our results monthly.

FGD, Staff H

#### Integration of Mobile Phone–Based Prevention of Mother-To-Child HIV Transmission Cascade Analysis Tool Into the Existing Health System

Integration is the extent to which a strategy can be integrated within an existing system [42]. A key recommendation for future implementation and successful integration of the mPCAT as part of SAIA was to orient and train all those who are part of the PMTCT care cascade on mPCAT and SAIA and ensure that there is clear communication to participants on expectations related to facility-level system improvements. FGD participants from 1 facility explained that although all those who were aware of the intervention were supportive of making changes to workflow and patient flow, it was challenging to get involvement and acceptance of proposed changes among those who were not trained on the mPCAT and SAIA.

## Discussion

### Principal Findings

Improvements to the mPCAT’s interface, including changes to its appearance, navigation, and input fields, were made during usability testing. Acceptability and adoption of the mPCAT was high, with users describing the app easy to use, both for entering and interpreting data. All 3 facilities in the feasibility study used the mPCAT to inform system improvement decision making in delivering Option B+ and reported that this process increased communication across service teams at the health facility.

### Implications for Practice, Scale-Up, and Future Research

Usability testing revealed that a simplified interface would be easier for first-time users to enter the data without errors. This adaptation integrated the important user-centered design principle of universal design. That is, the product should be accessible to as many people as possible, with particular attention to the user’s level of technological literacy [43]. In this case, as most of the study participants had relatively low technological literacy, having a single data entry field per page, which had to be filled before being able to advance to the next page, assisted in focusing the user’s attention and minimizing data entry errors, especially missing data entry fields.

Several important implications for successful mHealth app development and implementation were identified through the usability and feasibility testing. The presence of software developers during usability testing was very helpful in developing a more usable app, allowing them to see firsthand the priorities and challenges of end users and understand the reality of their work environments. Although the participants were able to provide feedback on what was confusing or difficult about the app, they were often unsure of how the app could be changed to be more usable. Having software developers help facilitate the usability test meant that they were able to offer several options for changes or add features that the participants could respond to, options that participants often would not have known to suggest themselves. Similarly, software developers were able to observe users and identify issues that users might not have flagged themselves. For example, developers observed that participants often skipped data entry inputs, which would lead to errors in the results. Hence, the adapted app was revised such that each item would need to be answered before a user could advance to the next item, improving error recovery. In addition, software developers were able to observe and immediately troubleshoot bugs and error messages that users encountered, rather than expecting nontechnical users to describe the issue from a distance. This collaborative process between users and software developers was critical in the development of a more user-centered, empathic design [44,45]. It also allowed for the development of an app that was locally contextualized, an important factor in the acceptance and successful adoption of mHealth projects [32,33].

Software updates to mPCAT also included simplifying coding so that minor changes could be updated by nontechnical persons. In the early version of the mPCAT app, coding for data entry items and results were scattered throughout the code, making it difficult for any nondeveloper to find the codes to change a data entry item. This was addressed by reformatting the code to pull all questions and formulas from a static JavaScript Object Notation (JSON) file, creating a Web-based graphical user interface that allows someone to provide a list of data entry fields and formulas that converts back to a JSON format that the app requires. Thus, future changes such as changing the data entry questions from English to another language, adding new items to the cascade, and altering formulas used to calculate results can be done by a nondeveloper.

Simplified coding and use of open-access software are 2 strategies that can ensure ease of adaptation of the mPCAT to local contexts such as changing the language of the app (eg, from English to Portuguese) or revising data entry fields to match national data collection forms. Currently, the original PCAT in Microsoft Excel has been or is in the process of being adapted to various other chronic care cascades in SSA, such as pediatric HIV (Kenya), hypertension (Mozambique), major mental health disorders (Mozambique), family planning uptake for people living with HIV (Kenya), preexposure prophylaxis uptake by at-risk youth (Kenya). It is anticipated that other app versions of the cascade analysis tool will be developed in the future. The mPCAT for PMTCT services is also being used in the SAIA-scale trial (R01MH113435) in 1 province in Mozambique, serving a population of approximately 2 million inhabitants.

Software applications, including mHealth apps, require continuous updating. National data collection forms change, health information systems change (eg, District Health Information System 2 [DHIS2]), phone technologies change, and therefore, apps need to constantly evolve to adapt to these changes. Changes to keep mHealth apps relevant require long-term funding, a challenge, given limited funding opportunities for long-term mHealth projects [32]. This is further challenged with common scenarios where mHealth apps are free and have limited advertising potential to sustain its own funding. In addition, the limited availability of a skilled electronic health and mHealth workforce in SSA [32,46-48] is also a critical gap that needs investment to ensure the continued success and sustainability of mHealth in strengthening health systems and improving health outcomes. Similarly, given the high turnover of health workers in SSA [49], ongoing staff training on the mPCAT, both independently and within the broader context of systems analysis and improvement approaches such as SAIA, is needed.

By harnessing the availability and power of mobile phones, frontline health workers were able to immediately, directly, and tangibly use their clinical documentation and routinely reported data to inform improvements to their own practice and workflow in their health facility. It is not uncommon in SSA, where there is a critical health workforce shortage [49], for health workers to dedicate significant time on data collection, entry, and reporting without active engagement in data use [50,51]. In addition, few mHealth technologies currently exist to support health workers’ use of data to inform decision making about HIV services [52]. The mPCAT tool and findings from this study substantially contribute to minimizing this critical gap between frontline health worker and independent data use for decision making.

### Limitations

There are limitations to this study. First, care should be taken when generalizing these findings to health settings beyond the study sites. The number of participants was small and although participation across PMTCT service delivery points (ANC, maternity, and children at-risk care) was secured, it was not representative of all health care providers who offer Option B+ services in Kenya and Mozambique. However, the purpose and benefit of feasibility studies is to assess how an intervention or strategy is implemented in uncontrolled, real-world settings, to inform future effectiveness trials, implementation, and scale-up [32,35,42]. Second, self-reported measurement of mHealth app use can be biased; a more objective measure would be to collect usage data on frequency and duration from the app itself, a feature to consider for future app updates. However, during FGDs and IDIs, participants described specific examples of how the mPCAT was used to inform system improvements that triangulated the self-reported use data. Third, although this study followed up 3 months post-implementation with users, sustained use of the mPCAT post-study period was not evaluated. However, sustained use is being studied as part of a trial evaluating the effectiveness of SAIA (R01MH113435), of which mPCAT is an integral part, when administered and managed by the Ministry of Health. Through this study, the mPCAT is being used by health workers in all health facilities in Maniça Province with PMTCT services.

### Conclusions

The collaborative process between software developer and user led to the development of an optimally usable and feasible mPCAT tool that was easy to use and easy to integrate into work routines. The mPCAT allows health workers and managers to quickly, clearly, and autonomously identify system inefficiencies in the delivery of Option B+ services and make data-driven decisions for system improvements. Findings from this study are critical in informing the development of a future controlled trial to determine the effectiveness of the mPCAT in reducing PMTCT dropout rates and contribute to the elimination of new pediatric HIV infections in SSA.

## Abbreviations

ANC: antenatal care
ART: antiretroviral therapy
CQI: continuous quality improvement
FGD: focus group discussion
HEI: HIV-Exposed Infants
IDIs: In-depth interviews
JSON: JavaScript Object Notation
mHealth: mobile health
mPCAT: mobile phone–based prevention of mother-to-child HIV transmission cascade analysis tool
PCAT: prevention of mother-to-child HIV transmission cascade analysis tool
PMTCT: prevention of mother-to-child HIV transmission
SAIA: system analysis and improvement approach
SSA: sub-Saharan Africa
